# Design, analysis, and presentation of crossover trials

**DOI:** 10.1186/1745-6215-10-27

**Published:** 2009-04-30

**Authors:** Edward J Mills, An-Wen Chan, Ping Wu, Andy Vail, Gordon H Guyatt, Douglas G Altman

**Affiliations:** 1 Faculty of Health Sciences, Simon Fraser University, Burnaby, Canada; 2 Mayo Clinic, Mayo School of Medicine, Rochester, USA; 3 Department of Epidemiology, London School of Hygiene & Tropical Medicine, London, UK; 4 School of Medicine, The University of Manchester, Manchester, UK; 5 Department of Clinical Epidemiology & Biostatistics, McMaster University, Hamilton, Canada; 6 Centre for Statistics in Medicine, Oxford University, Oxford, UK

## Abstract

**Objective:**

Although crossover trials enjoy wide use, standards for analysis and reporting have not been established. We reviewed methodological aspects and quality of reporting in a representative sample of published crossover trials.

**Methods:**

We searched MEDLINE for December 2000 and identified all randomized crossover trials. We abstracted data independently, in duplicate, on 14 design criteria, 13 analysis criteria, and 14 criteria assessing the data presentation.

**Results:**

We identified 526 randomized controlled trials, of which 116 were crossover trials. Trials were drug efficacy (48%), pharmacokinetic (28%), and nonpharmacologic (30%). The median sample size was 15 (interquartile range 8–38). Most (72%) trials used 2 treatments and had 2 periods (64%). Few trials reported allocation concealment (17%) or sequence generation (7%). Only 20% of trials reported a sample size calculation and only 31% of these considered pairing of data in the calculation. Carry-over issues were addressed in 29% of trial's methods. Most trials reported and defended a washout period (70%). Almost all trials (93%) tested for treatment effects using paired data and also presented details on by-group results (95%). Only 29% presented CIs or SE so that data could be entered into a meta-analysis.

**Conclusion:**

Reports of crossover trials frequently omit important methodological issues in design, analysis, and presentation. Guidelines for the conduct and reporting of crossover trials might improve the conduct and reporting of studies using this important trial design.

## Introduction

Because they reduce bias associated with imbalance in known and unknown confounding variables, randomized clinical trials (RCTs) represent the 'gold standard' for evaluating therapeutic effectiveness.[1] Unlike the parallel group trial, crossover trials provide each participant with two or more sequential treatments in a random order usually separated by a washout period [2]. Within a trial, each participant is able to act as his or her own control and permits between and within group comparisons [3,4].

For the study of new and developmental drugs, crossover studies are extremely popular [4,5], particularly when the new treatment may only be a slight modification to the standard. In this case, there is likely to be a positive correlation in the responses to the new and old treatments making the crossover design ideal [6]. Crossover studies are most appropriate in studies where the effects of the treatment(s) are short-lived and reversible and are best suited to trials related to symptomatic but chronic conditions or diseases [3,7]. It is generally agreed that the crossover design should not be used when the condition of interest is unstable and may change regardless of interventions [3]. In spite of criticism [8], however, the crossover design appears to be used commonly in inappropriate circumstances [3,9].

Despite their popularity, little is known about the quality or prevalence of randomized crossover trials. We aimed to review key methodological issues in the reporting of these trials in a representative sample of published trials.

## Methods

### Study cohort

Our study is nested within a larger analysis of RCTs [10] where we used an extended version of the Cochrane search strategy (phase 1) to identify all randomized trials published in December 2000 and indexed on PubMed by July 2002 [11]. A randomized trial was defined as a prospective study assessing health-care interventions in human participants who were randomly allocated to study groups. Abstracts were initially screened to exclude obvious non-trials, and complete primary reports in the languages AWC could read (English and French) were reviewed for all remaining studies.

We defined randomized crossover trials as studies where an individual receives two or more interventions through randomization to one of a set of prespecified sequences of treatments. Appendix 1 displays common characteristics and features of crossover trials. We included crossover trials of any intervention in any health condition. We excluded studies examining primarily cost-effectiveness or diagnostic test properties, as well as studies employing re-randomization which involves randomization of study participants into the second stage of a clinical trial [1].

### Data collection

Data extraction was conducted by two independent reviewers (PW and EM) using a standardized pre-piloted form. We classified trials by journal type, specialty, and intervention. We also recorded the trial design, study aim, number of groups (interventions, periods), number of data collection sites, funding sources, and sample size. If information about funding sources and number of study sites was unclear from the trial report, we requested clarification from the trialists. We assessed the reporting of several important methodological details. We recorded descriptions of sample size calculations and primary outcomes. With liberal definitions of adequacy [12], the reporting of patient preference and methods of random sequence generation, and allocation concealment were recorded. We also noted the handling of non-compliers, carryover, period, and treatment effects. We calculated descriptive summary statistics both overall and stratified by study design. We entered the data into an electronic database such that duplicate entries existed for each study; when two entries did not match, we reached consensus through discussion and 3^rd ^party arbitration (BR).

### Data analysis

In order to assess inter-rater reliability on inclusion of articles, we calculated a kappa score which provides a measure of inter-rater agreement independent of chance [5]. We determined the proportion of crossover trials for each item reported using simple tabulations and calculated the exact confidence intervals around a proportion [13].

## Results

### Results of our literature search

In total, 519 randomized trials published in December 2000 were identified. Of these, 116 or 22% were identified as crossover studies. Of the 116 publications included, 2 reported 3 separate trials [14,15], and 7 reported two independent trials within their publication [16-22]. Therefore, we included a total of 127 randomized crossover trials. Agreement on the final cohort was excellent (K= 0.94).

### Characteristics of the individual trials

In total, 30/127 (24%) trials measured drug pharmacokinetics, 36/127 (28%) were non-drug interventions while almost half, 61/127 (48%) were studies of drug efficacy. The number of periods ranged between 1 and 6, as six trials reported only on the first period. The median sample size was 15 (interquartile range: 8–38). Additional File 1 details the reporting characteristics of included studies stratified by study design (drug efficacy vs. pharmacokinetic vs. non-drug intervention). Of all 116 included publications, one was a letter to the editors [23], one was a summary of previously conducted research [24], and one did not contain an abstract [25]. Of the remaining trials, 77/113 (68%) used the term "crossover" in their title or abstract while 36/113 (33%) did not.

### Design of the individual trials

Several important study design characteristics were poorly reported (Additional File 1). For example, while 92/127 (72%) trials employed an AB/BA design (2 periods, 2 treatments), the study design was unclear in approximately a quarter of studies, 29/127 (23%). In almost three-quarters of included studies, carryover effects were not addressed in the methods section, 90/127 (71%), although 87/127 (70%) studies either used or explained the absence of a washout period. In 37/127 (30%) of studies it was unclear whether washout was considered. In the majority of studies, 114/127 (90%), it was not reported how groups were randomized, while allocation concealment was reported in less than a fifth of trials, 22/127 (17%). In total, sample size calculations before the study were provided in 26/127 (20%) studies. Of these, 8/26 (31%) reported using a paired data design and, 5/26 (19%, 95% CI: 9–38%) reported post-hoc power calculations in their results.

### Analysis of the individual trials

One hundred and seventeen trials (117/127 (92%) adequately detailed the handling of attrition. Of these, 74/117 (63%) reported applying an intention-to-treat (ITT) approach, whereby all patients randomized are included in the analysis. Tests for carryover and period effects were described or used in 22/127 (17%) and 17/127 (13%) of all included studies respectively. While the test for treatment effect was adjusting for co-variates in 4/127 (3%) studies, 121/127 (95%) studies reported a paired analysis.

Almost all studies 109/127 (86%) did not provide details regarding patient flow. Only 15/127 (12%) studies adequately described this component in their study design, with only 3/127 (2%) trials providing the CONSORT patient flow diagram recommended for parallel group trials.

Patient preference regarding intervention was reported in 10/127 (92%) of the studies. Individual participant data were presented in 15/127 (12%) studies while results were displayed graphically in 25/127 (20%). A paired summary statistic was reported in 118/127 (93%) of studies. Although the CI or SE was reported in 38/127 (29%) studies, it was calculable in most of the remaining studies that had not reported it, 78/89 (88%). Finally, in 79/127 (62%) of the studies, the trialists based their analysis and conclusions on the differences between groups as opposed to differences within individuals (i.e. within groups) – the latter was reported in only 3/127 (2%) studies. Interestingly, in 45/127 (35%) studies, the authors interpreted their results based on both differences within and between groups.

## Discussion

We found that important design issues are often under-reported in randomized crossover trials. Given their popularity – representing almost a quarter of trials published in December 2000 [10] – few reported important methodological issues such as allocation concealment, issues of carryover effects, and within-participant effects. Transparency and interpretation can be improved by creating standard reporting guidelines for authors and journals reporting the cross-over trial design. As yet the CONSORT reporting guidelines [12] have not been extended specifically for crossover trials.

There are several important strengths and limitations to be considered in our analysis. Strengths include our rigorous searching of PubMed during the study period, ensuring that adequate time had passed to allow all potential trials to be filed on the database. We extracted data in duplicate to reduce abstraction errors and resolved discrepancies by consensus. There are also limitations to consider. We searched only PubMed, the largest and most accessed database of medical articles. Other databases may have included additional articles. While every randomized trial published in December 2000 was read and appraised, it is possible that we missed some trials originally designed as crossover trials that were reported as parallel trials, reporting on only the first or second period of the trial. The methodological issues that we examined are a matter of debate. While evidence of bias exists for methodological issues such as blinding, sequence generation and allocation concealment [26], such evidence is lacking for other details such as flow diagrams, patient preference, and importantly, carryover effects. It is possible that if we had identified other methodological issues, we would have found different results. However, we developed these criteria based on studies in which we have participated and widespread consensus on methodological criteria, as reported in the CONSORT Statements [27]. Our data abstraction focused on prespecified criteria. During peer-review, a reviewer noted the important issue of differing analysis issues according to whether the main outcome measure in a trial is continuous, categorical, ordinal or binary, issues we had not considered. Finally, our analysis is based on the assumption that the reporting of methods and results in a published article reflects what was actually done. It is possible that some authors did conduct the methodological item, but failed to report it [28].

The crossover design has numerous advantages that investigators may wish to use for early stage trials. The particular strength of this design is that the interventions under investigation are evaluated within the same patients and so eliminates between-subject variability [4]. Further, this trial design permits opportunities of head-to-head trials and patients receiving multiple treatments can express preferences for or against particular treatments.

However, even when properly applied, crossover trials may have certain weaknesses. Patients may drop out after the first intervention period and thus not receive a second or third treatment. This makes within-subject comparison impossible [3] and is particularly important if withdrawal is related to side-effects [2,7]. This further complicates the concept of intent-to-treat analysis as patients randomized may complete the first period, but randomization typically does not occur at the second period. Also, there may be a residual [5] or carry-over of effect of treatments across study periods, which could potentially distort the results obtained during the second treatment or subsequent periods [7,29], although examples of this are few [30]. Thus, the observed treatment effects will depend upon the order in which they were received.

Some have argued against consistent testing for carryover effects of interventions across periods as carry-over effects are rare and statistical manipulation after the fact cannot address the impact of a carry-over effect.[30] Senn, in particular, has argued for a common sense approach to crossover trials, where no carry-over is assumed and thus, not tested for.[31] He specifically argues that tests for carry-over are generally underpowered even with an appreciable carry-over effect. He recommends instead that the wash-out period between periods be sufficient to prevent carryover effects. This paper does not aim to solve this issue, but rather displays the incongruence across crossover trials on the issue of carry-over and other design issues.

Another major potential threat to the validity of the crossover design involves the use of inappropriate statistical analysis [2]. Given that subjects act as their own controls, the analyses could be based on paired data (using an unpaired test) [5,6] and the within-subject variability in outcomes could be considered in sample size calculations [32]. Essentially, the use of a paired design is much more efficient than a parallel group design when researchers expect a high correlation between patients' responses to the different treatments.

## Conclusion

We found large heterogeneity in the reporting of crossover trials, possibly reflecting a lack of standards within the field. There is a clear need for minimum standards for transparent reporting of crossover trials.

## Abbreviations

RCTs: Randomized Clinical Trials; CONSORT: Consolidated Standards of Reporting Trials.

## Competing interests

The authors declare that they have no competing interests.

## Authors' contributions

AWC, AV, EM, DA, GG contributed to study concept.

AWC conducted the searches.

AWC, EM, PW conducted data abstraction.

AWC, EM, PW analyzed the data.

AWC, EM, PW, DA, GG wrote initial drafts of the manuscript.

AWC, AV, EM, PW, DA, GG approved the final manuscript.

## Appendix 1

Features used to assess reporting of methodological details in published crossover studies

### Design

Carryover: concept recognized in the methods section, credibly was absent and washout was either used or explained absence.

Allocation: Randomization and concealment methods are described.

Sample size calculation: methods reported and explained (prospective versus retrospective, paired vs. unpaired analysis)

### Analysis

Non-Compliers: clear if all participants are included, excluded, included under intention to treat (ITT) or not mentioned

Test for carryover effect: Yes formal, Yes informal, No, or unclear

Test for period effect: Yes formal, yes informal, no, not clear

Test for treatment effect: paired/unpaired, adjusted/unadjusted for period effect

Patient preference recorded: yes or no

### Presentation

Patient flow: presented as a CONSORT style diagram or other method

Detail for primary outcome:

• Individual data presented: Yes versus no

Inference

• paired summary statistic: Yes, No but calculable, No

Slant of paper: authors base slant of paper on differences between groups, within in groups or a combination

**Figure 1:**
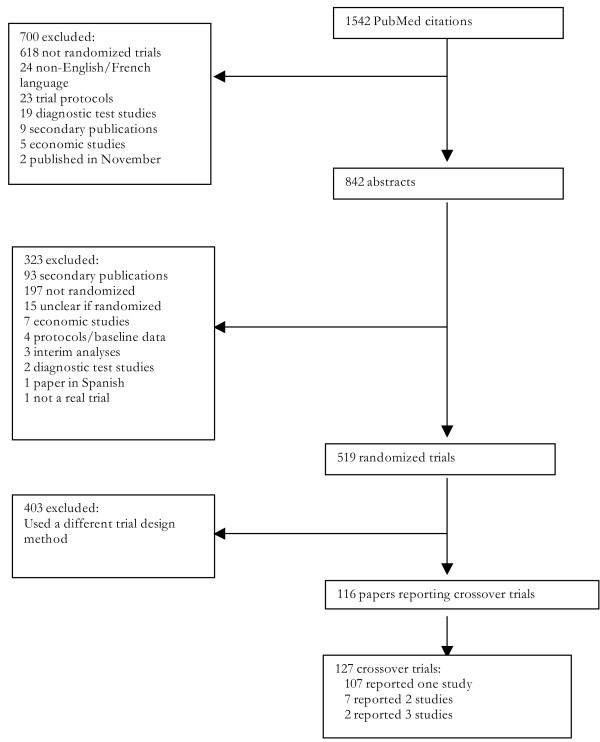
**Flow chart of included studies**.

## Supplementary Material

Additional file 1 **Reporting characteristics of included crossover studies stratified by study setting (drug efficacy vs. pharmacokinetic vs. non-drug intervention)** Click here for file
