# Supplementary material for: Design, analysis, and presentation of crossover trials
Source: Trials. 2009 Apr 30;10:27. doi: 10.1186/1745-6215-10-27 (PMC2683810; doi:10.1186/1745-6215-10-27)
Supplement: Additional file 1 — Reporting characteristics of included crossover studies stratified by study setting (drug efficacy vs. pharmacokinetic vs. non-drug intervention) [file 1745-6215-10-27-S1.doc]

Additional File 1: Reporting characteristics of included crossover studies stratified by study setting (drug efficacy vs. pharmacokinetic vs. non-drug intervention)

|  | **Setting** | | | | |
| --- | --- | --- | --- | --- | --- |
| **Characteristics** | **Drug Efficacy**  **(n = 61)** | **Pharmacokinetic**  **(n = 30)** | | **Non-Drug Intervention**  **(n = 36)** | **TOTAL** |
| **AB/BA design** | | | | | |
| Yes | 44 (72%) | 23 (77%) | | 25 (70%) | 92 |
| Other design | 3 (5%) | 0 (0%) | | 3 (8%) | 6 |
| Not clear | 14 (23%) | 7 (23%) | | 8 (22%) | 29 |
| **Carryover concept recognized in methods** | | | | | |
| Yes | 15 (25%) | 12 (40%) | 9 (25%) | | 36 |
| No | 46 (75%) | 18 (60%) | 27 (75%) | | 91 |
| **Washout** | | | | | |
| Used, or explained absence | 42 (69%) | 25 (83%) | 20 (56%) | | 87 |
| Not used but absence unexplained | 1(1%) | 0 (0%) | 2 (5%) | | 3 |
| Not mentioned | 18 (30%) | 5 (17%) | 14 (39%) | | 37 |
| **Randomization** | | | | | |
| Explained explicitly | 7 (11%) | 0 (0%) | 6 (17%) | | 13 |
| Not explicitly | 54 (89%) | 30 (100%) | 30 (83%) | | 114 |
| Not clear | 0 (0%) | 0 (0%) | 0 (0%) | | 0 |
| **Allocation Concealment** | | | | | |
| Yes | 16 (26%) | 2 (7%) | 4 (11%) | | 22 |
| No | 45 (74%) | 28 (93%) | 31 (89%) | | 104 |

| **Sample size calculation** | | | | |
| --- | --- | --- | --- | --- |
| Yes | 14 (23%) | 8 (27%) | 4 (11%) | 26 |
| No | 47 (77%) | 22 (73%) | 32 (88%) | 101 |
| Post hoc power calculation in results | 2 (3%) | 1(3%) | 2 (6%) | 5 |
| **Appropriate component of**  **variance used in sample size calculation** | | | | |
| Yes | 6 (10%) | 2 (7%) | 0 (0%) | 8 |
| No | 55 (90%) | 28 (93%) | 36 (100%) | 119 |
| **Non-compliers in analysis** | | | | |
| Not mentioned | 6 (10%) | 0 (0%) | 4 (11%) | 10 |
| Excluded | 24 (39%) | 10 (33%) | 9 (25%) | 43 |
| ITT | 31 (51%) | 20 (66%) | 23 (64%) | 74 |
| **Test for carryover effect** | | | | |
| Yes | 11 (18%) | 5 (17%) | 6 (17%) | 22 |
| No | 45 (74%) | 23 (77%) | 27 (75%) | 95 |
| Not clear | 5 (8%) | 2 (6%) | 3 (8%) | 10 |
| **Test for period effect** | | | | |
| Yes | 8 (13%) | 5 (17%) | 4 (11%) | 17 |
| No | 46 (75%) | 22 (74%) | 29 (81%) | 97 |
| Not clear | 7 (12%) | 3 (10%) | 3 (8%) | 13 |
| **Test for treatment effect** | | | | |
| Adjusted | 2 (3%) | 2 (7%) | 0 (0%) | 4 |
| Unadjusted | 48 (79%) | 24 (80%) | 29 (81%) | 101 |
| Not clear for period effect | 11 (18%) | 4 (13%) | 7 (19%) | 22 |
| **Test for treatment effect** | | | | |
| Paired | 57 (93%) | 30 (100%) | 35 (97%) | 121 |
| Unpaired | 4 (7%) | 0 (0%) | 1 (3%) | 6 |
| **Patient preference recorded** | | | | |
| Yes | 7 (11%) | 0 (0%) | 3 (8%) | 10 |
| No | 54 (89%) | 30 (100%) | 33 (92%) | 117 |
| **Patient flow included** | | | | |
| CONSORT diagram | 2 (3%) | 0 (0%) | 1(3%) | 3 |
| No | 50 (82%) | 27 (90%) | 32 (89%) | 109 |
| Study design | 9 (15%) | 3 (10%) | 3 (8%) | 15 |
| **All patients accounted for** | | | | |
| Yes | 31 (51%) | 20 (67%) | 23 (64%) | 74 |
| No | 24 (39%) | 10 (33%) | 9 (25%) | 43 |
| Not clear | 6 (10%) | 0 (0%) | 4 (11%) | 10 |
| **Individual data presented** | | | | |
| Yes | 7 (11%) | 5 (17%) | 3 (8%) | 15 |
| No | 54 (89%) | 25 (83%) | 33 (92%) | 112 |
| **Graphs with individual data presented** | | | | |
| Yes | 13 (21%) | 6 (20%) | 6 (17%) | 25 |
| No | 48 (79%) | 24 (80%) | 30 (83%) | 102 |
| **1^st^ period analyzed as a separate trial** | | | | |
| Yes | 5 (8%) | 1 (3%) | 0 (0%) | 6 |
| No | 56 (92%) | 29 (97%) | 36 (100%) | 121 |
| **Paired summary statistic presented** | | | | |
| Yes | 54 (89%) | 30 (100%) | 34 (94%) | 118 |
| No but calculable | 3 (4%) | 0 (0%) | 1 (3%) | 4 |
| No | 4 (7%) | 0 (0%) | 1(3%) | 5 |
| **CI or SE for paired summary statistic presented** | | | | |
| Yes | 15 (25%) | 17 (57%) | 6 (17%) | 38 |
| No, but calculable | 40 (66%) | 11 (37%) | 28 (78%) | 79 |
| No | 6 (9%) | 2 (6%) | 2 (5%) | 10 |
| **Interpretation/conclusions of paper based on:** | | | | |
| Difference between groups | 34 (58%) | 26 (87%) | 19 (53%) | 79 |
| Within-participant | 2 (1%) | 0 (100%) | 1(3%) | 3 |
| Both | 25 (41%) | 4 (13%) | 16 (44%) | 45 |
